# Structural and Functional Dysbiosis of Fecal Microbiota in Chinese Patients With Alzheimer's Disease

**DOI:** 10.3389/fcell.2020.634069

**Published:** 2021-02-04

**Authors:** Zongxin Ling, Manlian Zhu, Xiumei Yan, Yiwen Cheng, Li Shao, Xia Liu, Ruilai Jiang, Shaochang Wu

**Affiliations:** ^1^State Key Laboratory for Diagnosis and Treatment of Infectious Diseases, Collaborative Innovation Center for Diagnosis and Treatment of Infectious Diseases, National Clinical Research Center for Infectious Diseases, School of Medicine, The First Affiliated Hospital, Zhejiang University, Hangzhou, China; ^2^Department of Geriatrics, Lishui Second People's Hospital, Lishui, China; ^3^Institute of Hepatology and Metabolic Diseases, Hangzhou Normal University, Hangzhou, China; ^4^Department of Liver Diseases, Institute of Translational Medicine, The Affiliated Hospital of Hangzhou Normal University, Hangzhou, China; ^5^Department of Intensive Care Unit, School of Medicine, The First Affiliated Hospital, Zhejiang University, Hangzhou, China

**Keywords:** Alzheimer's disease, *Bifidobacterium*, *Faecalibacterium*, gut-brain axis, sequencing

## Abstract

Increasing evidence suggests that gut dysbiosis plays vital roles in a variety of gut–brain disorders, such as Alzheimer's disease (AD). However, alterations of the gut microbiota as well as their correlations with cognitive scores and host immunity have remained unclear in well-controlled trials on Chinese AD patients. In this study, samples from 100 AD patients, and 71 age- and gender-matched, cognitively normal controls were obtained to explore the structural and functional alterations of the fecal microbiota targeting the V3–V4 region of the 16S rRNA gene by MiSeq sequencing, and to analyze their associations with clinical characteristics. Our data demonstrated a remarkably reduction in the bacterial diversity and alterations in the taxonomic composition of the fecal microbiota of the AD patients. Interestingly, the abundant butyrate-producing genera such as *Faecalibacterium* decreased significantly, where this was positively correlated with such clinical indicators as the MMSE, WAIS, and Barthel scores in the AD patients. On the contrary, abundant lactate-producing genera, such as *Bifidobacterium*, increased prominently, and were inversely correlated with these indicators. This shift in the gut dysbiosis of the microbiota, from being butyrate producers to lactate producers, contributed to immune disturbances in the host that could be used as non-invasive biomarkers to distinguish the controls from the AD patients. Moreover, several predicted functional modules, including the biosynthesis and the metabolism of fatty acids, that were altered in the microbiota of the AD patients could be utilized by the bacteria to produce immunomodulatory metabolites. Our study established the structural and functional dysbiosis of fecal microbiota in AD patients, and the results suggest the potential for use of gut bacteria for the early, non-invasive diagnosis of AD, personalized treatment, and the development of tailor-made probiotics designed for Chinese AD patients.

## Introduction

Alzheimer's disease (AD) is an age-related neurodegenerative disease characterized by slowly progressive memory decline and cognitive dysfunction, and no preventative or disease-modifying treatments are available for it at present (Gaugler et al., 2019). AD is a leading cause of dementia, and its prevalence is increasing drastically among aging populations worldwide. According to the World Alzheimer Report 2015, almost 47 million people worldwide were expected to be affected by dementia in 2015, with 9.9 million new diagnoses each year. This number is estimated to exceed 130 million by 2050, with the greatest increase expected in low- and middle-income countries. In 2017, 121,404 deaths caused by AD were recorded in USA, making it the sixth leading cause of death among Americans of all ages and the fifth leading cause among elderly Americans (age ≥65 years) (Gaugler et al., 2019). In addition, the economic costs of the disease are formidable due to the care needed by the growing number of patients with AD and other dementias. Thus, addressing the rapidly growing incidence of AD should be regarded as a global public health priority.

Numerous studies in recent decades have focused on elucidating the etiopathology of AD, but its pathogeneses remain unclear, and no therapeutic strategy is available to cure this disease. The most important risk-related factor for AD is advancing age, and as lifespans increase and demographic aging occurs worldwide, the number of AD patients is expected to increase drastically. The depositions of amyloid-beta peptide, a product of the cleavage of the amyloid-beta protein precursor, and the abnormal tau protein can be used as diagnostic markers for AD (Holtzman et al., 2011). However, whether they are the causes of AD or its consequences remains unknown. Many recent studies have noted that several infectious agents, such as *Chlamydia pneumoniae*, herpes simplex virus type 1, and several types of spirochaete and fungi, are involved in the pathogenesis of late-onset AD (De Chiara et al., 2012; Alonso et al., 2014; Balin and Hudson, 2014; Itzhaki, 2014; Miklossy, 2015; Pisa et al., 2015). This has prompted the suggestion that long-term, largely subclinical pathogenic infection might contribute to the characteristic neurodegeneration that occurs due to AD (Balin and Hudson, 2014). Based on these findings, Reis et al. (2010) considered AD itself as an infectious disease. Recent advances have revealed that microbiota of the human gut have numerous beneficial functions, such as immune development and resistance to pathogens, and serve as an important reservoir of pathogenic bacteria, viruses, and fungi. Previous work has clarified the role of gut microbiota in regulating multiple neuro-chemical pathways through the gut–brain axis (Bonfili et al., 2017). The dysbiosis of intestinal microbiota might impair intestinal mucosal integrity, increase intestinal permeability, and then disturb the intestinal homeostasis, where this can contribute to spreading potential pathogens to the target organs, such as the brain. Several studies have found altered gut microbiota in AD patients, which suggests that gut microbiota may be involved in AD pathogenesis (Vogt et al., 2017; Zhuang et al., 2018; Liu P. et al., 2019). Our group has observed that the transplantation of probiotics, prebiotics, and even fecal microbiota can ameliorate cognitive deficits and neurodegeneration in a model of mice with AD through the modulation of the gut microbiota (Sun et al., 2019a,b; Sun et al., 2020). Thus, the eubiosis of gut microbiota might have beneficial roles in preventing the occurrence and development of AD and other gut–brain disorders. However, the changing patterns of the composition and diversity of the gut microbiota are not always uniform in AD patients, but vary with population, geographical location, diet, and habits. The difference in the genetic background of hosts and dietary constitutions between Western and Chinese populations might contribute to the baseline disparity in the composition of the microbiota between them, which might in turn influence the roles of specific bacteria in the etiopathology of AD.

Lishui is a city that features dense mountains with a vegetation coverage of 80.79%, and has ranked second on China's ecological index behind Zhejiang for each of the past 13 years. It is also called the longevity town of China, with nearly 200 centenarians. The average life expectancy of the residents of Lishui is 80.06, 2.76 years higher than the national average. Its heredity, dietary patterns, and natural geographical environment influence the health of and incidence of diseases among the population, and may also influence the overall structure and function of the people's gut microbiota. The higher depth of sequencing and coverage with the advent of advanced sequencing techniques have made it possible to decipher key unknown functional taxa in Chinese AD patients. In this study, the fecal microbiota associated with AD are analyzed in a large AD cohort and matched healthy controls from Lishui by using the 16S rRNA high-throughput gene MiSeq platform, and are correlated with clinical indicators to provide novel targets for the early, non-invasive diagnosis and personalized treatment of AD as well as the development of tailor-made probiotics designed for Chinese AD patients.

## Methods

### Subjects' Enrollment

A total of 100 well-controlled Chinese AD patients, who were diagnosed based on the criteria of the National Institute of Neurological and Communicative Diseases and Stroke/AD and Related Disorders Association, were recruited from Lishui, Zhejiang province (China) from February 2019 to November 2019, with 71 cognitively normal subjects as control. The cognitive and functional status were scored using the Mini-Mental State Examination (MMSE, Chinese version), the current version in the Wechsler Adult Intelligence Scale series (WAIS-IV, published in 2008), and instrumental Barthel activities of daily living. Each participant was scanned on magnetic resonance imaging (MRI), with AD patients diagnosed as brain atrophy. The detailed demographic data and medical history (such as hypertension, diabetes mellitus, hypercholesterolemia, coronary heart disease, diarrhea, and constipation) were collected using a set of questionnaire. The exclusion criteria included: family history of dementia; any kind of other neurodegenerative disease such as Parkinson's disease; confirmed mental illness such as schizophrenia; any kind of tumor; antibiotic, prebiotic, probiotic, or synbiotic administration in the previous month; known active infections such as viral, bacterial, or fungal infections; other diseases such as inflammatory bowel disease, irritable bowel syndrome, or other autoimmune diseases. These protocols for the study were approved by the Ethics Committee of Lishui Second People's Hospital (Zhejiang, China) and written informed consent was obtained from each of the subject or their guardian before enrollment.

### Fecal Sample Collection and DNA Extraction

Approximately 2 g of a fresh fecal sample was collected in a sterile plastic cup, and stored at −80°C after preparation within 15 min until use. Bacterial genomic DNA was extracted from 300 mg of homogenized feces using a QIAamp® DNA Stool Mini Kit (QIAGEN, Hilden, Germany) according to the manufacturer's instructions, with additional glass-bead beating steps on a Mini-beadbeater (FastPrep; Thermo Electron Corporation, Boston, MA, USA). The amount of DNA was determined using a NanoDrop ND-1000 spectrophotometer (Thermo Electron Corporation); the integrity and size were checked by 1.0% agarose gel electrophoresis containing 0.5 mg/ml ethidium bromide. All DNA was stored at −20°C before further analysis.

### Amplicon Library Construction and Sequencing

Amplicon libraries were constructed with Illumina sequencing-compatible and barcode-indexed bacterial PCR primers 341F (5′-CCTACGGGNGGCWGCAG-3′)/785R (5′-ACTACHVGGGTATCTAATCC-3′), which target the V3–V4 regions of the 16S rRNA gene (Fadrosh et al., 2014). All PCR reactions were performed with KAPA HiFi HotStart ReadyMix using the manufacturer's protocol (KAPA Biosystems) and ~50 ng of extracted DNA per reaction. Thermocycling conditions were set at 95°C for 1 min, 55°C for 1 min, then 72°C for 1 min for 30 cycles, followed by a final extension at 72°C for 5 min. All PCR reactions were performed in 50 μl triplicates and combined after PCR. The amplicon library was prepared using a TruSeq™ DNA sample preparation kit (Illumina Inc, San Diego, CA, USA). Prior to sequencing, the PCR products were extracted with the MiniElute® Gel Extraction Kit (QIAGEN) and quantified on a NanoDrop ND-1000 spectrophotometer (Thermo Electron Corporation) and Qubit 2.0 Fluorometer (Invitrogen). The purified amplicons were then pooled in equimolar concentrations and the final concentration of the library was determined by Qubit (Invitrogen). Negative DNA extraction controls (lysis buffer and kit reagents only) were amplified and sequenced as contamination controls. Sequencing was performed on a MiSeq instrument (Illumina) using a 300 × 2 V3 kit together with PhiX Control V3 (Illumina) (Ling et al., 2019; Liu X. et al., 2019). MiSeq sequencing and library construction were performed by technical staff at Hangzhou KaiTai Bio-lab.

### Bioinformatic Analysis

The 16S rRNA gene sequence data set generated from the MiSeq run were first merged and demultiplexed into per samples using the QIIME version 1.9.0 with default parameters (Caporaso et al., 2010). Chimera sequences were detected and removed using the USEARCH software based on the UCHIME algorithm (Edgar et al., 2011). Open-reference operational taxonomic unit (OTU) pick was then performed with USEARCH V7 referenced against Greengenes database version 13.8 at 97% sequence similarity (Edgar, 2010; Mcdonald et al., 2012). OTUs with a number of sequences <0.005% of the total number of sequences were discarded as recommended (Navas-Molina et al., 2013). The result was an OTU table, which was used for subsequent downstream analysis.

For taxonomic assignment, the most abundant sequences were chosen as the representative sequences of corresponding OTUs. Taxonomic assignment of individual datasets were classified against the Greengenes database version 13.8 using both RDP classifier and UCLUST version 1.2.22 methods implemented in QIIME (Wang et al., 2007; Edgar, 2010). Any sequences that were identified as members of Eukarya, Archaea, Mitochondria, Chloroplasts, and Cyanobacteria lineages, were removed. Alpha diversity was calculated with QIIME software with Python scripts base on the sequence similarity at 97% level, including index of observed OTUs, abundance-based coverage estimator (ACE), Chao1 estimator, Shannon, Simpson, Evenness, and PD whole tree. Sequence coverage was assessed in mothur by rarefaction curves and Good's coverage (Good, 1953; Schloss et al., 2009). Beta diversity was measured by jaccard, bray-curtis, unweighted UniFrac, and weighted UniFrac distance calculated with 10 times of subsampling by QIIME. These distances were visualized by principal coordinate analysis (PCoA) (Lozupone and Knight, 2005). Hierarchical clustering was performed and heatmap was generated using a Spearman's rank correlation coefficient as a distance measure and a customized script developed in the R statistical package. The output file was further analyzed using Statistical Analysis of Metagenomic Profiles software package (STAMP) version 2.1.3 (Parks et al., 2014).

For the predictive functional analyses, PiCRUSt software package version 1.0.0 was used to identify predicted gene families and associated pathways from inferred metagenomes of taxa of interest identified from the compositional analyses, which was based on the fact that phylogeny and function are closely linked (Langille et al., 2013). Predicted functional genes were categorized into Clusters of Orthologous Groups (COG) and into Kyoto Encyclopedia of Genes and Genome (KEGG) orthology (KO), and compared across patient groups using STAMP. Pathways and enzymes were assigned using KEGG database options built into the pipeline. The pathways that were non-prokaryotic, had fewer than 2 sequences in each cohort, or had a difference in mean proportions <0.1% were excluded from analysis. The characterization of microorganismal features differentiating the gastric microbiota was performed using the linear discriminant analysis (LDA) effect size (LEfSe) method (http://huttenhower.sph.harvard.edu/galaxy/) for biomarker discovery, which emphasizes both statistical significance and biological relevance (Segata et al., 2011). With a normalized relative abundance matrix, LEfSe uses the Kruskal-Wallis rank sum test to detect features with significantly different abundances between assigned taxa and performs LDA to estimate the effect size of each feature. A significant alpha at 0.05 and an effect size threshold of 3 were used for all biomarkers discussed in this study.

Correlation analysis was performed using sparse compositional correlation (SparCC) algorithm on the complete OTU table collapsed to the genus level, which was introduced by Friedman and Alm and was known for its robustness to the compositional effects that are influenced by the diversity and sparsity of correlation in human microbiome data sets (Friedman and Alm, 2012). SparCC was employed to represent co-abundance and co-exclusion networks between OTUs. For SparCC, 1000 bootstrap replicates were used to calculate significance values, and considered correlation coefficients greater or <0.2 and −0.2, respectively, and *p* < 0.05. This set of iterative procedures were applied separately to normal, peritumor and tumor data sets to infer the basis correlation values within and/or between paired sampling sites. Visualization of the network was achieved using Cytoscape version 3.4.1.

### Systemic Inflammatory Cytokines Analysis

Serum samples from these participants were obtained using their fasting blood in the early morning. Using a 27-plex magnetic bead based immunoassay kit (Bio-Rad, CA, USA), the following cytokines were quantified: interleukin-1β (IL-1β), IL-1 receptor antagonist (IL-1ra), IL-2, IL-4, IL-5, IL-6, IL-7, IL-8, IL-9, IL-10, IL-12(p70), IL-13, IL-15, IL-17, Eotaxin, Fibroblast growth factor-basic (FGF-basic), granulocyte colony-stimulating factor (G-CSF), granulocyte-macrophages colony-stimulating factor (GM-CSF), interferon gamma (IFN-γ), interferon gamma-inducible protein 10 (IP-10), monocyte chemotactic protein-1 (MCP-1), macrophages inflammatory protein-1α (MIP-1α), platelet-derived growth factor (PDGF-bb), MIP-1β, regulated upon activation normal T-cell expressed and secreted (RANTES), tumor necrosis factor-alpha (TNF-α), and vascular endothelial growth factor (VEGF). The Bio-Plex 200 system was utilized for the analysis of Bio-Rad 27-plex human group I cytokines and the Bio-Plex assay (Bio-Rad) was performed according to the manufacturer's directions. The results expressed as picogram per milliliter (pg/mL) using standard curves integrated into the assay and Bio-Plex Manager v5.0 software with reproducible intra- and inter-assay CV values of 5–8%.

### Statistical Analysis

White's non-parametric *t*-test, independent *t*-test, or Mann-Whitney *U*-test were applied for continuous variables. Pearson chi-square or Fisher's exact test were used for categorical variables between groups. Spearman's rank correlation test was utilized for correlation analyses. Statistical analysis was performed using the SPSS v19.0 (SPSS Inc., Chicago, IL) and STAMP v2.1.3 (Parks et al., 2014). R packaged and GraphPad Prism v6.0 were used for preparation of graphs. All tests of significance were two sided, and *p* < 0.05 or corrected *p* < 0.05 was considered statistically significant.

### Accession Number

The sequence data from this study are deposited in the GenBank Sequence Read Archive with the accession number SRP262626.

## Results

### Subject Characteristics

Table 1 shows the characteristics of the Chinese AD patients as well as the age- and gender-matched cognitively normal, healthy controls. There were no significant differences in terms of gender, body mass index, smoking, drinking and comorbidities of hypertension, hypercholesterolemia, diabetes mellitus, and coronary heart disease between the healthy controls and the AD patients (*p* > 0.05), while the MMSE, WAIS, and Barthel scores were clearly lower in Chinese AD patients than in the healthy controls (*p* < 0.05).

**Table 1:** Characteristics of the participants.

**Parameters**	**AD patients (*n* = 100)**	**Healthy controls (*n* = 71)**	***p***
Age (y)	74.14 ± 9.21	73.11 ± 7.75	0.105
Gender (male/female)	43/57	35/36	0.415
BMI (Mean ± SD)	22.12 ± 3.45	23.45 ± 3.32	0.164
Smoking, no.	4	3	0.942
Drinking, no.	2	1	0.772
Antibiotics use, no.	0	0	
Complications, no.			
Hypertension	37	25	0.445
Diabetes mellitus	17	11	0.793
Hypercholesterolemia	18	10	0.495
Coronary heart disease	15	8	0.481
Diarrhea	2	3	0.395
Constipation	7	5	0.991
Cognitive and functional status			
MMSE score	4.27 ± 6.06	27.21 ± 2.04	<0.01
WAIS score	35.31 ± 15.35	90.14 ± 10.04	<0.01
Barthel score	23.22 ± 23.15	76.75 ± 7.79	<0.01

*BMI, body mass index; SD, standard deviation; no, numbers; MMSE, Mini-Mental State Examination; WAIS, Wechsler Adult Intelligence Scale*.

### Altered Overall Structure of the Fecal Microbiota in AD

**Figure 1:**
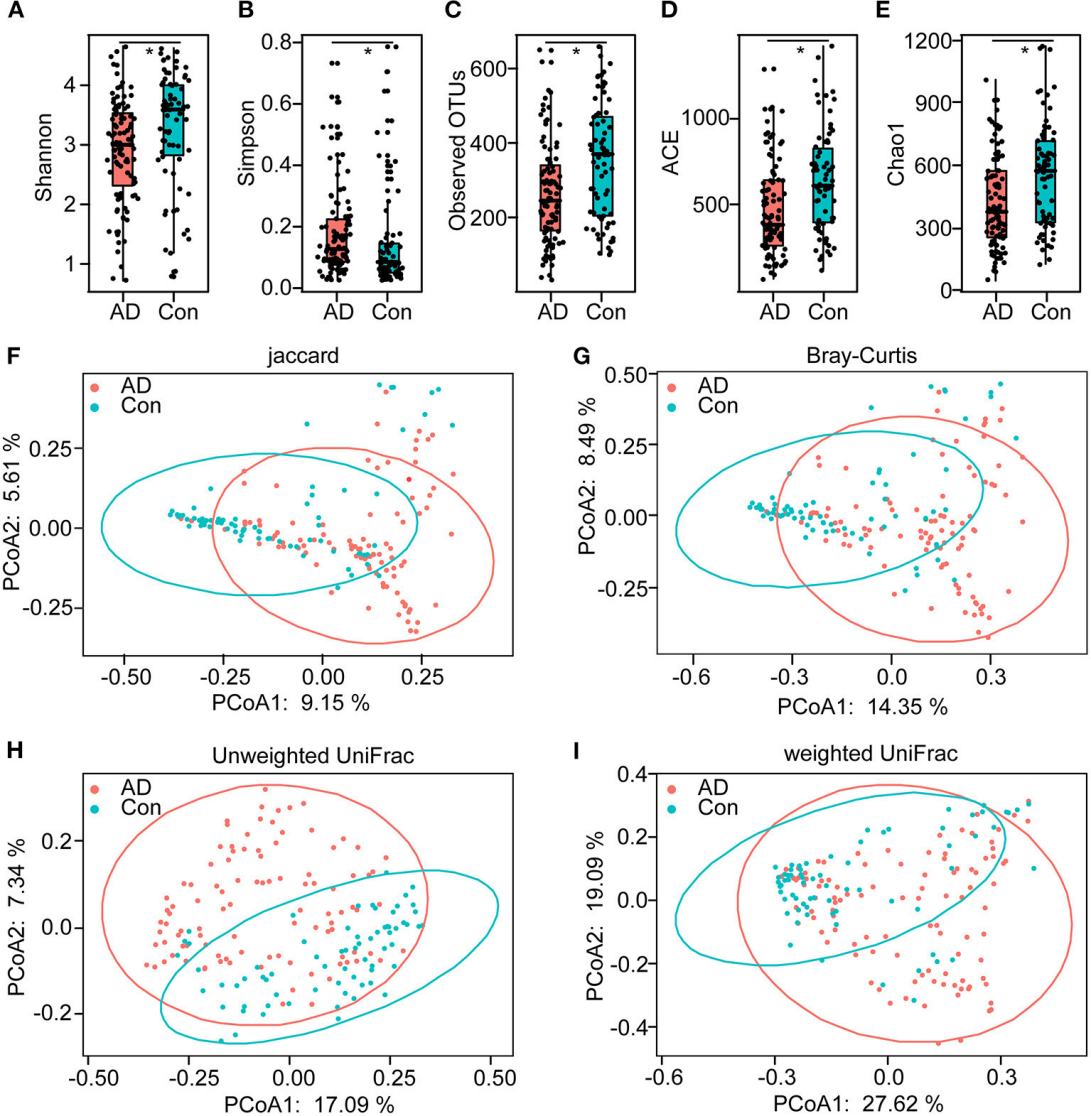
The altered bacterial diversity and richness of the fecal microbiota in Chinese AD patients. The diversity indices of Shannon **(A)** and Simpson **(B)**, and the richness indices of the observed OTUs **(C)**, ACE **(D)**, and Chao1 **(E)** were used to evaluate the overall structure of the fecal microbiota in the AD patients and the healthy controls. The data are presented as mean ± standard deviation. Unpaired *t*-tests (two tailed) were used to analyze the variation between the groups. Principal coordinate analysis (PCoA) plots of individual fecal microbiota based on Jaccard **(F)**, Bray–Curtis **(G)**, and unweighted **(H)** and weighted **(I)** UniFrac distances in the Chinese AD patients and the healthy controls. Each symbol represents a sample. *p < 0.05.

In total, 5,760,348 high-quality reads (2,421,229 reads of the controls and 3,339,119 of AD patients), with an average of 33,686 reads per sample, were obtained for the subsequent analysis of the microbiota. The value of Good's coverage was 99.24%, indicating that a majority of bacterial phylotypes (2,366 OTUs) in the fecal microbiota had been identified. Interestingly, the alpha-diversity indices, including Shannon's and Simpson's indices, were significantly different between the controls and the AD patients, indicating decreased bacterial diversity in AD-associated microbiota (Figures 1A,B). Richness indices, including the observed OTUs, ACE, and Chao1, were also significantly higher in the controls than in the AD patients (Figures 1C–E). Despite significant inter-individual variations, the PCoA based on the Jaccard, Bray–Curtis, unweighted UniFrac, and weighted UniFrac algorithms also divided the two groups into different clusters (Adonis test: *p* < 0.01; Figures 1F–I). Thus, the alpha- and beta-diversity analyses demonstrated that the overall structure of the AD-associated fecal microbiota had changed significantly compared with that of the controls.

### Composition of Changed Fecal Microbiota in AD Patients

**Figure 2:**
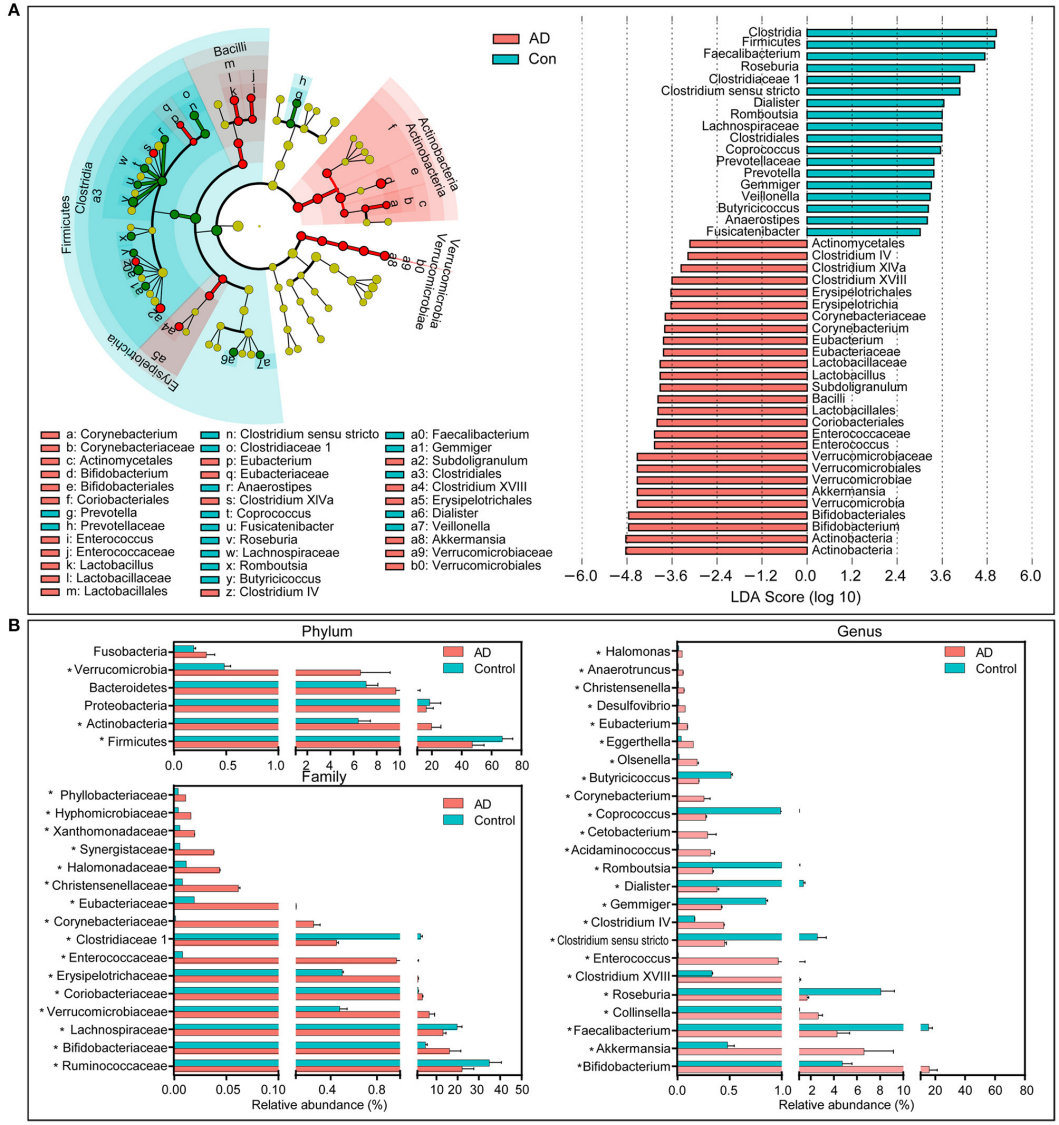
Differential bacterial taxa between the Chinese AD patients and the healthy controls. The LEfSe identified the taxa with the greatest differences in abundance between the Chinese AD patients and the healthy controls. Only the taxa meeting a significant LDA threshold value of >3 are shown **(A)**. Comparisons of the relative abundance of the abundant bacterial taxa at the level of bacterial phylum, family, and genus **(B)**. The data are presented as the mean ± standard deviation. Mann–Whitney *U*-tests were used to analyze variation between the Chinese AD patients and the healthy controls. *p < 0.05 compared with the control group.

The compositions of the fecal microbiota in the AD patients and the controls were assessed at different taxonomic levels. Using the RDP classifier, the sequences were classified as 10 phyla, 76 families, and 203 genera. The distribution of the phyla and the genera are shown in Supplementary Figures 1, 2, respectively, and suggested significant inter-personal variations. By using the LEfSe, our discriminant analyses showed that many key taxa were clearly different between the AD and the control group (LDA score >3, *p* < 0.05, Figure 2). Only bacterial phylotypes with an average relative abundance of more than 0.01% were selected here for the LEfSe. The representative cladogram demonstrated the dysbiosis of AD-associated fecal microbiota in the Chinese AD patients. Of these differential functional bacterial taxa, we found that Actinobacteria and Verrucomicrobia had clearly increased in the AD patients, while Firmicutes had significantly decreased at the phylum level. At the family level, 13 key functional bacterial families including *Bifidobacteriaceae, Verrucomicrobiaceae, Coriobacteriaceae, Erysipelotrichaceae, Enterococcaceae*, and *Corynebacteriaceae* had significantly increased in AD patients while three families—*Ruminococcaceae, Lachnospiraceae*, and *Clostridiaceae* 1—had drastically decreased. At the genus level, 24 key functional bacterial genera had changed significantly between the groups while only eight genera—*Faecalibacterium, Roseburia, Clostridium sensu stricto, Gemmiger, Dialister, Romboutsia, Coprococcus*, and *Butyricicoccus*—had decreased in AD patients. Supplementary Figure 3 shows the heatmap of the bacterial genera in the AD patients and the controls. It shows the relative percentages of most genera identified in each sample. Intriguingly, traditionally beneficial genera, such as *Bifidobacterium* and *Akkermansia*, had drastically increased in the Chinese AD patients.

**Figure 3:**
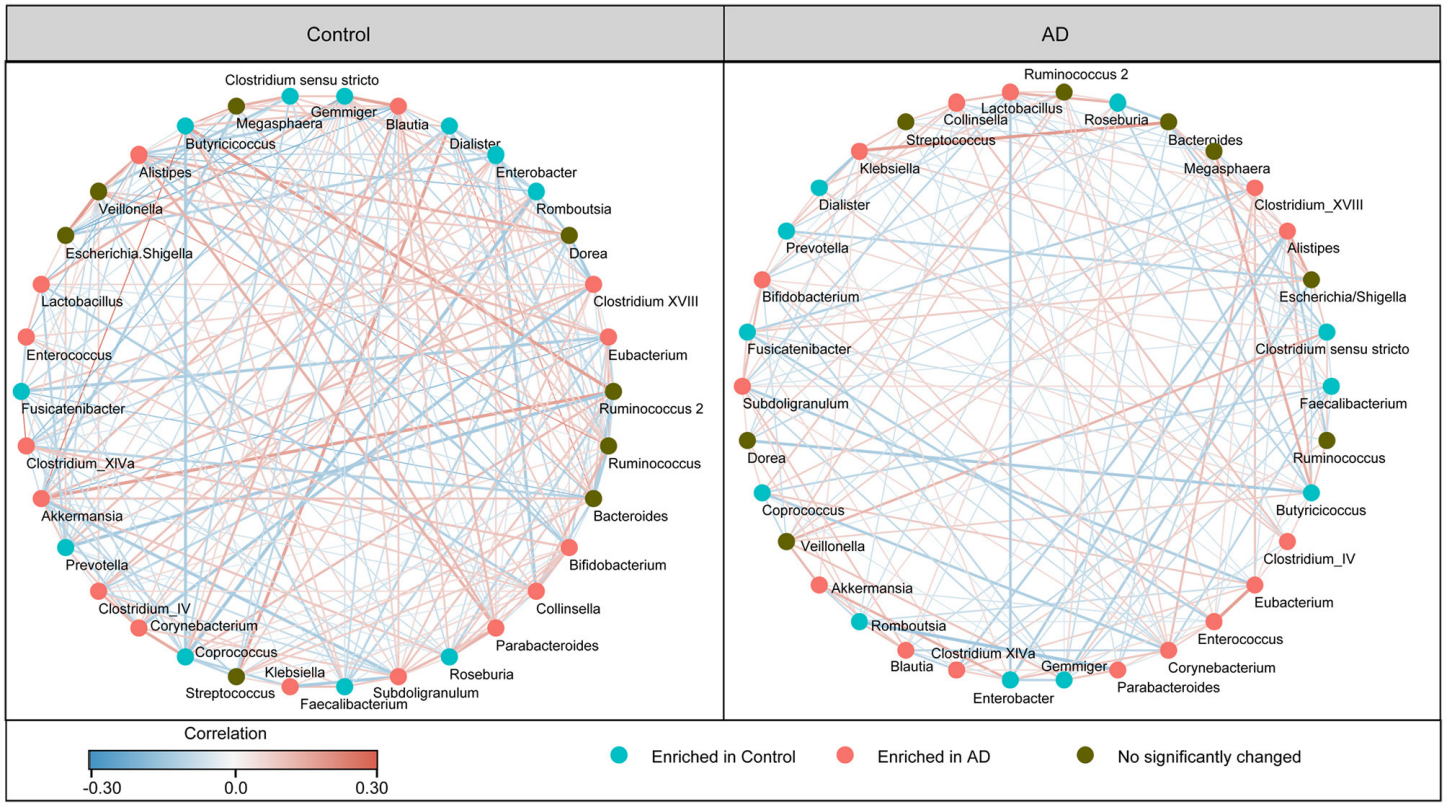
Strengths of the correlation between abundant fecal microbiota in the Chinese AD patients and the healthy controls. Correlation network of the abundant fecal microbiota in the healthy controls and the AD patients. The correlation coefficients were calculated with the Sparse Correlations for Compositional (SparCC) data algorithm. Cytoscape version 3.4.0 was used for network construction. The red and blue lines represent positive and negative correlations, respectively. The correlation network became simpler in AD patients.

In addition, the structure of the fecal microbiota was determined by dynamic interactions between these community members. Our SparCC algorithm with FDR adjustments was used to generate correlation-based networks of microbial interaction based on the relative abundance of OTUs between the groups (Figure 3). We found a more complex network of interactions in healthy controls than that in the AD patients. More positive and negative correlations among the bacteria were found in the healthy controls than in the AD patients. Our data indicate the structural dysbiosis of the AD-associated fecal microbiota in the AD patients.

### Fecal Microbiota-Based Signature Discriminated Healthy Controls From AD Patients

**Figure 4:**
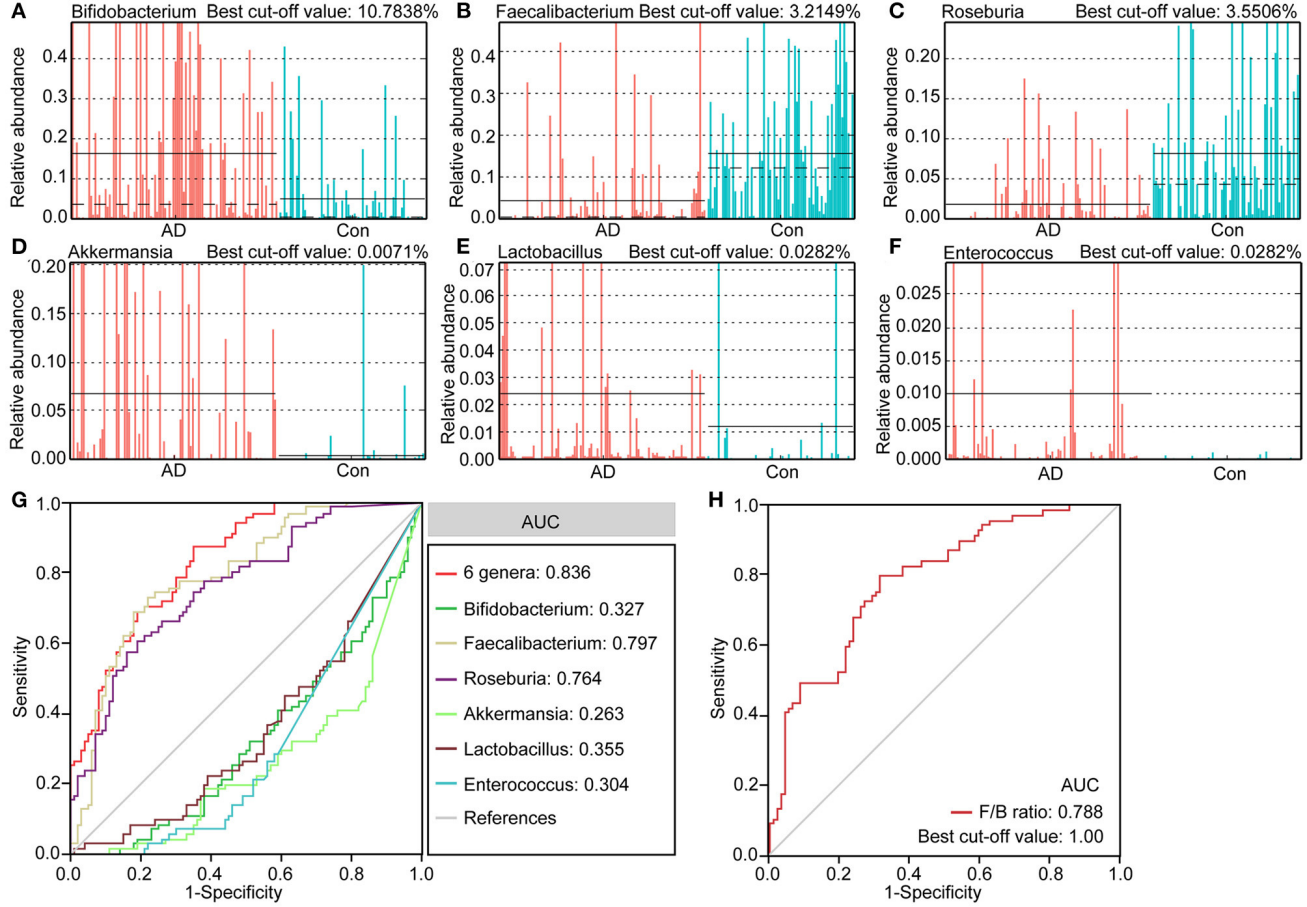
The differential genera as AD diagnostic markers. The relative abundance and best cut-off values of the differential genera such as Bifidobacterium **(A)**, Faecalibacterium **(B)**, Roseburia **(C)**, Akkermansia **(D)**, Lactobacillus **(E)**, Enterococcus **(F)** in each sample. Receiver operating characteristic (ROC) curves for the differential genera alone or in combination **(G)**, and Faecalibacterium/Bifidobacterium **(H)**, used to discriminate healthy controls from AD patients. AUC, the area under the receiver operating characteristic curve.

We identified several differential taxa in the AD-associated fecal microbiota. We then evaluated the value of using six abundant genera as biomarkers: *Bifidobacterium, Faecalibacterium, Roseburia, Akkermansia, Lactobacillus*, and *Enterococcus*. The differential features of these genera are shown in Figures 4A–F, which show significant inter-personal variations. We first used only one of the differential bacteria as predictor to generate the area under the receiving operating characteristic curves to obtain the area under the curve (AUC) ranging from 0.304 to 0.797 (Figure 4G). Figure 4 shows that enriched *Faecalibacterium* was the best discriminant predictor for the healthy controls (AUC: 0.797), with a best cut-off value of 3.2149%. Further, multivariable stepwise logistic regression analysis was applied to the list of AD-associated genera to determine the taxa that best distinguished the controls from the AD patients. We found that using all six abundant genera significantly improved predictive performance (AUC: 0.836). We also assessed the predictive value of the ratio of *Faecalibacterium/Bifidobacterium* (F/B ranged from 0.0001 to 2876.2660, Figure 4H). We found that the ratio of F/B could help discriminate between healthy controls and AD patients with an AUC of 0.788. Interestingly, the best cut-off value of the ratio of F/B was one. Therefore, these key differential genera can be used as potential biomarkers for discriminating between healthy controls and AD patients.

### Microbial Functional Dysbiosis in AD

**Figure 5:**
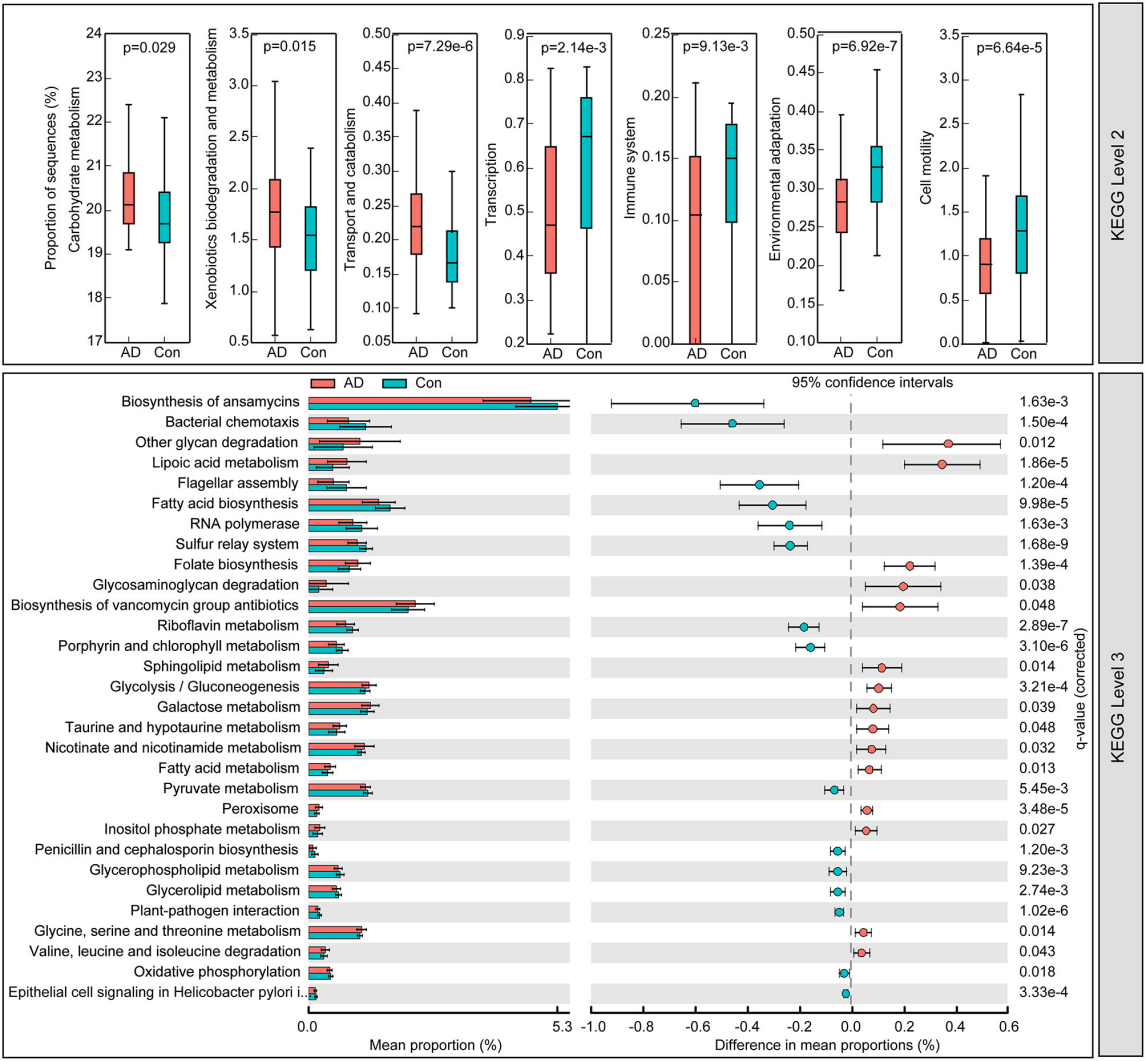
PiCRUSt-based examination of the fecal microbiome of the Chinese AD patients and the healthy controls. The different bacterial functions were evaluated between them based on two-sided Welch's *t*-test. Comparisons between the groups for each KEGG functional category (levels 2 and 3) are shown by percentage. The Benjamini–Hochberg method was used for multiple testing correction based on the false discovery rate (FDR) through STAMP.

To identify the metabolic and functional changes in the fecal microbiota between the AD patients and the controls, PiCRUSt was used to analyze the functional potential of the microbiota based on closed-reference OTU picking. We compared 64 KEGG pathways at level 2 and identified seven KEGG categories with clearly differential abundances between the AD patients and the controls. We found that carbohydrate metabolism, xenobiotics' biodegradation and metabolism, and transport and catabolism significantly increased in the AD patients, while transcription, immune system, environmental adaptation, and cell motility significantly decreased (*p* < 0.05; Figure 5). Specifically, 15 pathways in level 3, including the metabolism of fatty acids and lipoic acid, and folate biosynthesis, increased significantly, while 15 other pathways, including bacterial chemotaxis and the biosynthesis of fatty acid, decreased prominently in the AD-associated microbiota. Together, the functional dysbiosis of the fecal microbiota may participate in the pathogenesis and development of AD.

### Correlations Between Differential Genera, and Clinical Indicators and Host Immunity

**Figure 6:**
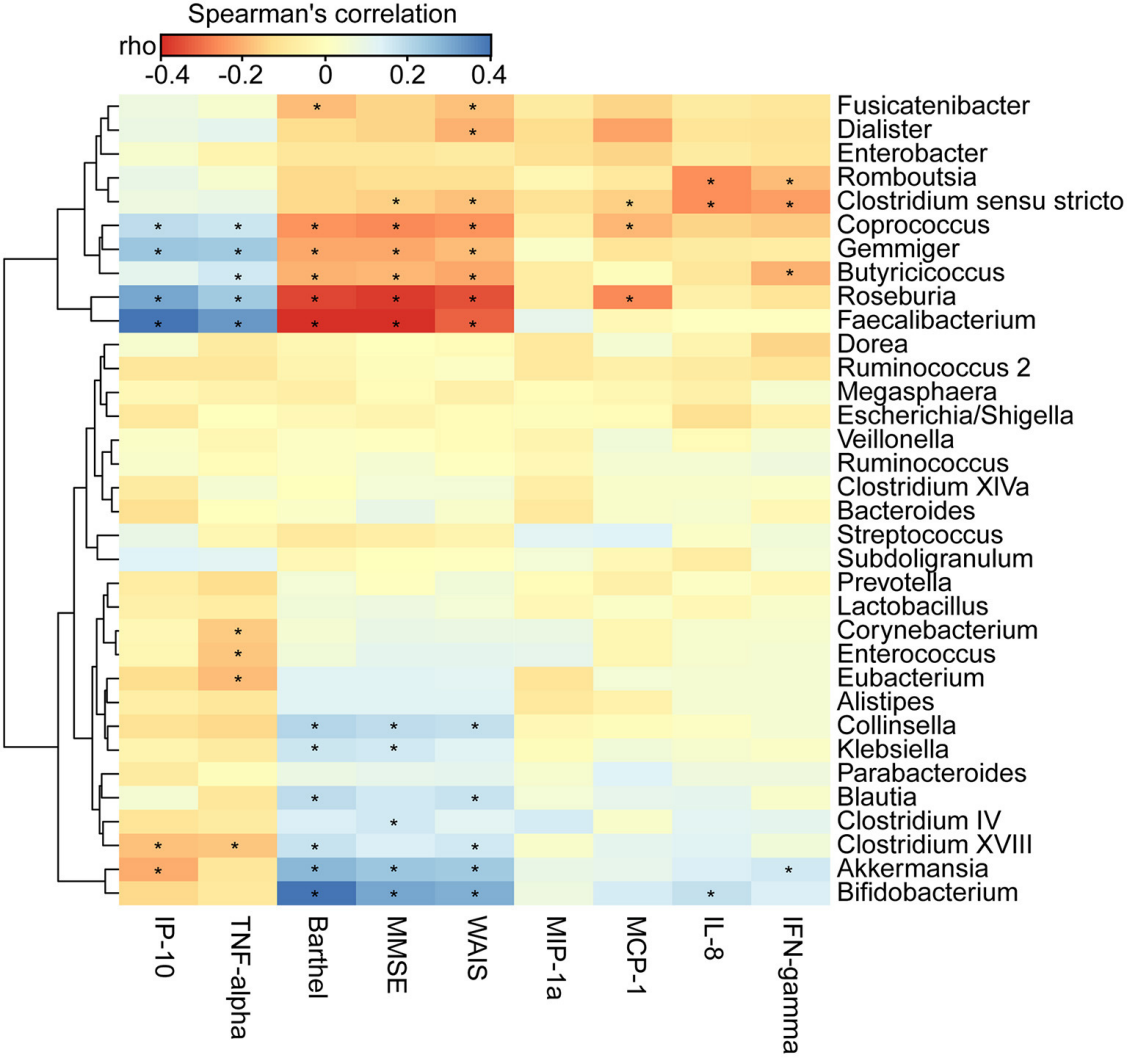
Correlation between fecal microbiota, and pro- and anti-inflammatory cytokines and chemokines and clinical indicators. The heatmap shows partial Spearman's correlation coefficients between 34 genera, and clinical indicators and host immunity in AD patients. Spearman's rank correlation (r) and probability (p) were used to evaluate statistical importance. *p < 0.05.

We found that the clinical indicators—the MMSE, WAIS, and Barthel scores—were significantly lower in AD patients (*p* < 0.01). By using the Bio-Plex Pro™ human cytokine group I panel 27-plex analysis, we observed that anti-inflammatory cytokines, such as IFN-γ, had significantly decreased, such pro-inflammatory cytokines as TNF-α had markedly increased, and several chemokines, such as IL-8, MCP-1, and MIP-1a, had clearly decreased. IP-10 had also decreased in the Chinese AD patients (Supplementary Figure 4; *p* < 0.05). To determine the associations between the deferential genera of the AD patients, and the clinical indicators and altered cytokines, we performed a correlation analysis using Spearman's rank correlation (Figure 6). Notably, such lactate producers as *Bifidobacterium* and propionate producers such as *Akkermansia* had the strongest negative correlations with clinical indicators such as MMSE, WAIS, and the Barthel scores, whereas butyrate-producing genera, such as *Faecalibacterium, Roseburia, Gemmiger, Coprococcus*, and *Butyricicoccus*, had positive correlations with the clinical indicators (*p* < 0.05). *Bifidobacterium* was negatively associated with IL-8, *Akkermansia* was negatively correlated with IFN-γ but positively correlated with IP-10, *Enterococcus* and *Corynebacterium* were positively correlated with the pro-inflammatory cytokine TNF-α, while *Faecalibacterium, Roseburia, Gemmiger*, and *Coprococcus* were negatively correlated with TNF-α and IP-10 (*p* < 0.05). Taken together, the enriched lactate-producing genera and the decreased butyrate-producing genera in the fecal microbiota of AD patients performed distinct roles in the progression of AD and differently modulated the immune response of the host. The altered fecal microbial profiles and their related host responses might be the key pathophysiology of AD.

## Discussion

In recent years, multi-omics techniques have revealed that the gut microbiota play a crucial role in promoting human health, as a result of which they are often referred to as the “forgotten organ.” Accumulating evidence indicates that the gut microbiota constitute a key factor in maintaining gut homeostasis by various complex mechanisms. Not only have the gut microbiota been invoked as a contributor to every gastrointestinal ailment, but the analyses of their influence have also been extended to other organs, such as the central nervous system (CNS). Exploring the roles and mechanisms of the gut microbiota in neurodegenerative diseases is an emerging field of research. Recently, several lines of research have suggested that changes in the composition and function of gut microbiota significantly affect neuronal function and, consequently, the host's behavior (Wang T. et al., 2015). The gut–brain axis of the microbiota has a proven role in regulating multiple neuro-chemical pathways. Microbiota–gut–brain axis signaling has uncovered a new era in psychiatry that is expected to provide novel targets for the diagnosis and treatment of psychiatric disorders and decipher their pathogeneses.

Aging is associated with an overstimulation of both innate and adaptive immune systems, resulting in a low-grade, chronic state of inflammation defined as inflammaging (Franceschi et al., 2000; Franceschi, 2007). This can increase gut permeability (“leaky gut”) and bacterial translocation (Ulluwishewa et al., 2011; Tran and Greenwood-Van Meerveld, 2013). As a major age-related neurodegenerative disorder, the onset of AD has been closely correlated with alterations in the gut microbiota (Vogt et al., 2017; Zhuang et al., 2018; Li et al., 2019; Liu P. et al., 2019; Wang et al., 2019). Most of these previous “AD microbiome” studies have been mainly conducted on a small scale of patients. In this study, 100 well-controlled Chinese AD patients and 71 age- and gender-matched normal controls were enrolled for AD microbiome analysis. In total, the deeper sequencing and higher coverage allowed us to identify low-abundance taxa in AD-associated fecal microbiota. In the results, structural changes in the fecal microbiota were evident in Chinese AD patients, with decreased alpha-diversity indices and altered beta-diversity ones. Inconsistent with previous clinical studies on AD patients, our study also indicated that Shannon significantly decreased in the AD patients (Liu P. et al., 2019), and such richness indices as the observed OTUs, ACE, and Chao1 were also significantly reduced (Vogt et al., 2017). Liu P. et al. (2019) have also shown significant compositional differences between AD patients and controls in PCoA plots based on Bray–Curtis dissimilarity, which were consistent with our study. Taken together, both alpha-diversity and beta-diversity indices provide powerful evidence of structurally dysbiotic AD microbiota in the AD patients.

In parallel with other similar studies, the structural dysbiosis observed in ours shows that the compositions of the AD-associated fecal microbiota also changed significantly. The distributions of bacterial taxa in the AD patients, at the levels of the phylum, family, and genus, were significantly different from those in healthy controls. An enrichment of the phyla Actinobacteria and Verrucomicrobia, and a decrease in the phylum Firmicutes were observed in AD-associated microbiota, while the amounts of such abundant phyla as Proteobacteria and Bacteroidetes did not change significantly, which is not consistent with previous studies (Zhuang et al., 2018; Liu P. et al., 2019). One aging indicator, the decreased ratios of Firmicutes/Bacteroidetes (Mariat et al., 2009), also did not change significantly in AD-associated microbiota. However, the ratios of Firmicutes/Actinobacteria were found to have decreased significantly (*p* < 0.05), which could reveal the bacterial dysbiosis in AD-associated microbiota. Several bacterial families—such abundant families as the *Ruminococcaceae, Lachnospiraceae*, and *Clostridiaceae* 1—decreased significantly in AD patients, while *Bifidobacteriaceae, Verrucomicrobiaceae, Coriobacteriaceae, Erysipelotrichaceae*, and *Enterococcaceae* increased significantly. *Ruminococcaceae* and *Lachnospiraceae* can produce different types of short-chain fatty acids (SCFAs). Among the SCFAs, butyrate has received particular attention in research owing to its beneficial effects on maintaining health. Butyrate can influence gastrointestinal physiology, the peripheral immunity of the liver metabolism, and the integrity of the blood–brain barrier, which can indirectly contribute to the functions of the brain (Fung et al., 2017). In addition, it can drive the maturation of the microglia, and is needed for the maintenance of mature microglia (Erny et al., 2015). However, the amounts of *Bifidobacteriaceae, Verrucomicrobiaceae, Coriobacteriaceae*, and *Enterococcaceae*, mainly lactate producers, increased in AD patients. Inconsistent with this study, Liu P. et al. (2019) found that the family *Enterobacteriaceae* is correlated with the presence and progression of AD, which can help distinguish between AD patients and healthy controls (AUC: 0.698). The changed fecal bacteria at the levels of phylum and family represent the dysbiosis of AD-associated fecal microbiota, but this is not suitable for a non-invasive diagnosis of AD using fecal bacteria at higher taxonomic levels.

In agreement with the altered bacteria at the family level, many such genera as the *Bifidobacterium, Akkermansia, Faecalibacterium, Collinsella*, and *Roseburia* and changed significantly in content the AD-associated fecal microbiota. Interestingly, traditionally beneficial bacteria, such as the *Bifidobacterium* and *Akkermansia*, increase in these AD patients while *Faecalibacterium* and *Roseburia* decrease significantly. Our ROC curves also show that these differential genera can be used as biomarkers to discriminate the controls from the AD patients, alone or together, which provides novel targets for a non-invasive diagnosis of AD. *Bifidobacterium*, mainly a lactate producer, is highly beneficial to humans, and has been used as a food supplement in dairy products (Camfield et al., 2011). An open-label, single-arm, preliminary clinical study conducted by Kobayashi et al. (2017, 2019) found that oral supplementation using *Bifidobacterium breve* A1 can improve the cognitive function and maintain the quality of life of the elderly by suppressing the gene expression of inflammation and immune-reactive genes. Different from previous studies on animals and clinical studies (Vogt et al., 2017), this traditionally beneficial genus was among the most abundant genera in the AD-associated fecal microbiota, which suggests that the *Bifidobacterium* genus may play a crucial role in the pathogenesis and development of AD. It is challenging to link species to 16S metagenomic data, but different species of *Bifidobacterium* may have different effects that can explain why *Bifidobacterium* spp. are commonly associated with healthy and diverse microbiota but sometimes also isolated in other conditions (Pineiro and Stanton, 2007). Thus, we needed to re-examine the therapeutic potential of *Bifidobacterium* in terms of maintaining cognitive function and treating dementia. Our data also showed that *Bifidobacterium* was significantly negatively correlated with the MMSE, WAIS, Barthel, and IL-8, which also shows that *Bifidobacterium* was not a beneficial genus in our clinical study. *Akkermansia*, a specialized mucin-degrading genus, can utilize mucin-derived sugars like fucose to produce propionate through the propanediol pathway (Ottman et al., 2017). Previous work has shown that *Akkermansia muciniphila* (typical strain) is associated with protection against obesity, enhancement of wound healing, augmented antitumor responses, and induced intestinal adaptive immune responses during homeostasis (Everard et al., 2013; Greer et al., 2016; Routy et al., 2018; Ansaldo et al., 2019). Combinations of *Akkermansia*, two strains of *Clostridium*, one strain of Eubacterium, one strain of Bifidobacterium, and inulin have recently been used as special synbiotics to treat type-2 diabetes mellitus. The discovery of *Akkermansia muciniphila* has opened new avenues for the use of this abundant intestinal symbiont in next-generation therapeutic products, and they can be used to target the dynamics of microbiota. Surprisingly, our data indicate that *Akkermansia* was among the most abundant genera in the AD-associated fecal microbiota. Similarly to *Bifidobacterium, Akkermansia* was negatively correlated with clinical indicators of AD, such as MMSE, WAIS, and Barthel, and anti-inflammatory cytokine such as IFN-γ. Based on our present observations, *Akkermansia* cannot always be considered a potentially beneficial bacterium, it might be harmful for the gut–brain axis in the context of the AD development in the elderly. Of these AD-enriched genera, *Clostridium* IV, *Desulfovibrio*, and *Corynebacterium* have been reported to be involved in the pathologic development of AD and other CNS diseases (Zhou et al., 2019), which is consistent with our findings. *Clostridium* IV was closely associated with type-2 diabetes and obesity in mice as well as the risk factors in AD development (Haan, 2006; Yamaguchi et al., 2016), which in turn is correlated negatively with the MMSE. Sawin et al. (2015) have also shown that *Desulfovibrio* can induce decreased levels of SCFAs that can influence pathologic conditions of CNS disease (Sampson et al., 2016). Previous studies have also found a decreased amount of *Corynebacterium* in patients suffering from depression and autism spectrum disorder (Strati et al., 2017; Yu et al., 2017), while *Corynebacterium* has been positively correlated with pro-inflammatory TNF-α. Different from Vogt's and Liu's studies, we found that such non-abundant genera as *Collinsella, Enterococcus, Olsenella, Eubacterium, Christensenella, Anaerotruncus*, and *Halomonas* were also enriched in AD-associated fecal microbiota (Vogt et al., 2017; Liu P. et al., 2019). *Collinsella*, one of the most abundant genera in the phylum Actinobacteria, was found to have increased prominently in the AD patients, but decreased in cases of relapsing–remitting multiple sclerosis (Chen et al., 2016), which was negatively correlated with MMSE, WAIS, and Barthel. *Enterococcus* (typical strain *E. faecalis*) can generate early Alzheimer-like neurofibrillary epitopes in primary rat cortical neurons (Underly et al., 2016), which can serve as harmful bacteria in AD etiopathology. Similarly to our findings, *Christensenella* was found to be increased in amounts in patients with Parkinson's disease in comparison with healthy controls (Petrov et al., 2017). These enriched AD-associated fecal genera, mainly lactate and propionate producers, may play a crucial role in the pathogenesis and development of AD.

However, reductions in the levels of *Faecalibacterium, Roseburia, Clostridium sensu stricto, Gemmiger, Dialister, Romboutsia, Coprococcus*, and *Butyricicoccus* were observed in AD-associated fecal microbiota. In particular, Biagi et al. (2016) and Wang F. et al. (2015) have shown that such butyrate producers as *Faecalibacterium, Roseburia*, and *Coprococcus* are negatively correlated with age. Our study found that these bacteria were positively correlated with AD clinical indicators, such as the MMSE, WAIS, and Barthel, and were negatively correlated with inflammatory cytokines, such as TNF-α and chemokines, such as IP-10. *Faecalibacterium* (typical strain *F. prausnitzii*), a major member of the Firmicutes phylum, is considered to be among the most important bacterial indicators of a healthy gut, and can modulate the inflammation of the level of the gut epithelium (Sokol et al., 2008). Beneficial *Faecalibacterium* has been found to be reduced in case of many intestinal disorders. Van Tongeren et al. (2005) observed a decreased relative abundance of *Faecalibacterium* in frail and elderly patients. In line with these findings, the decreased proportion of *Faecalibacterium* and increased *Bifidobacterium* have been found in elderly patients with Parkinson's disease (Scheperjans et al., 2015; Unger et al., 2016). All these changes may lead to a pro-inflammatory gut environment that may altogether lead to the chronic low-grade inflammation found in elderly persons with declining health. Previous studies have found that *Faecalibacterium* has anti-inflammatory properties due to its capability to produce butyrate and induce a tolerogenic cytokine profile (Sokol et al., 2008; Qiu et al., 2013), which can help extenuate these alterations in elderly AD patients. Liu J. et al. (2020) found that high-altitude Tibetan fermented milk can increase microbial diversity, and can elevate the levels of *Bacteroides* and *Faecalibacterium* in AD mice model, which are associated with cognitive improvements in mice afflicted with AD. The clinical comparative analyses and studies on animal mechanics confirm the beneficial roles of Faecalibacterium on mental health, which has prompted interest in considering this bacterium as a new-generation probiotic or psychobiotic. Gut *Roseburia* is part of commensal bacteria-producing SCFAs, especially butyrate, that affect immunity maintenance, colonic motility, and anti-inflammatory properties. The concomitant decreases in the well-known butyrate-producing bacterial genus *Roseburia* in many intestinal disorders (including type-2 diabetes, obesity, irritable bowel syndrome, nervous system conditions, and allergies), which suggests the potential of these bacteria as indicators of intestinal health (Tamanai-Shacoori et al., 2017). Consistently with our data, Keshavarzian et al. (2015) demonstrated the anti-inflammatory properties of *Roseburia*, and found that its levels are more abundant in feces of controls than in those of patients with Parkinson's disease. Neyrinck et al. (2011) found that the amount of *Roseburia* was inversely correlated with important markers of the metabolism and obesity of the host lipid. The role of *Roseburia* in protecting the nervous system from diseases has lately been highlighted, and has been shown to reduce neuroinflammation by regulating the gut–brain axis through its metabolite butyrate. *Coprococcus*, a less abundant bacterium in the large intestine, produces butyrate from fructose and propionate from lactate (via the acrylate pathway) (Reichardt et al., 2018). Together with Faecalibacterium, the butyrate-producing *Coprococcus* has been consistently associated with higher quality-of-life (QoL) indicators, which have been positively associated with several QoL scores (Valles-Colomer et al., 2019). Our previous study showed that *Coprococcus* is depleted in patients of depression (Jiang et al., 2015), even after correcting for the confounding effects of antidepressants (Valles-Colomer et al., 2019). Parashar and Udayabanu (2017) also found a reduction in fecal bacteria in the genus *Coprococcus* in patients with Parkinson's disease. Inconsistent with our microbiome study on AD patients here, Nagpal et al. (2019) found increased levels of *Coprococcus* in mild cognitive impairment participants in comparison with the controls. On the contrary, our study demonstrated that *Coprococcus* is positively correlated with clinical indicators of AD. *Butyricicoccus*, a butyrate-producing *Clostridium* cluster IV genus, was reduced in the feces of the AD patients. *Butyricicoccus* was found to be positively associated with the clinical indicators MMSE, WAIS, and Barthel, and anti-inflammatory cytokine IFN-γ, and negatively correlated with the pro-inflammatory cytokine TNF-α. Devriese et al. (2017) also found that reduced mucosa-associated *Butyricicoccus* activity in patients with ulcerative colitis was correlated with aberrant expressions of claudin-1, supporting its use as a pharmabiotic that preserves epithelial tight junction integrity. Zhang et al. (2017) also showed that the abundance of *Butyricicoccus* clearly decreases in a mouse model of AD in comparison with age-matched controls. Shen et al. (2019) observed that the regulation of gut microbiota by using silibinin and silymarin, especially with an increase in *Butyricicoccus*, might prohibit AD. Another *Clostridium* cluster I genus, *Clostridium sensu stricto*, was found to have decreased in AD patients (Vogt et al., 2017). *Clostridium sensu stricto* was positively associated with HDL and negatively associated with VLDL particles (Vojinovic et al., 2019), which are associated with a decreased risk of cardiovascular disease and stroke (Holmes et al., 2018). *Gemmiger*, also an SCFAs-producing genus, was positively related with the Montreal Cognitive Assessment scale score in patients with post-stroke cognitive impairment (Liu Y. et al., 2020). Our study also found that decreased levels of *Gemmiger* were positively associated with the MMSE, WAIS, and Barthel, and negatively correlated with inflammatory cytokines such as TNF-α and chemokines such as IP-10. Consistent with our study, Vogt et al. (2017) found that the genus *Dialister* (belonging to *Veillonellaceae*) was less abundant in AD participants, and exhibited the strongest correlations with such AD biomarkers of cerebrospinal fluid as Aβ42/Aβ40, p-tau, and p-tau/Aβ42. Therefore, all these decreased levels of AD-associated fecal genera, interacting with the AD-enriched genera, where this contributed to shifts in the SCFAs, might have participated in the pathogenesis and development of AD.

The level of endogenous SCFAs is influenced by many factors, of which gut bacterial metabolism is the most important. The dysbiosis of microbiota in patients of AD can change the balanced levels of SCFAs in the human body, while abnormal levels of SCFAs may negatively affect human health. Zhao et al. (2018) have claimed that the SCFAs serve as the bridge within this associations among diet, intestinal microbiota, and health. We observed that several metabolic pathways, such as those for carbohydrate metabolism, xenobiotics biodegradation and metabolism, and the immune system, changed significantly in AD-associated fecal microbiota. The characteristics of the AD microbial profiles changed from butyrate producers, such as Faecalibacterium into lactate producers, such as *Bifidobacterium*. These alterations contributed to shifts in metabolic pathways from butyrate to lactate, which might have participated in the pathogenesis of AD. However, the specific roles of the AD-associated signatures and their functions should be explored in further studies.

Our study is limited in some ways. First, it used the 16S rRNA amplicon rather than metagenomic sequencing, which limited us to the finding of specific bacteria related to AD at the species level. Second, our cross-sectional study investigated only healthy controls and confirmed AD participants. To decipher the dynamic interplay between microbiota and AD, a longitudinal follow-up case-control study should include different stages of AD, such as the preclinical stage and the mild cognitive impairment stage, that signify the transition from health to AD. Third, the fecal microbial signatures and the corresponding metabolites as well as the non-invasive diagnostic model associated with AD still need a larger sample size of clinical studies to be validated. Fourth, culturomics should be used to obtain the AD-associated bacteria, and animal experiments can help determine the cause–effect relationship between these bacteria and the pathogenesis of AD.

In summary, we found altered bacterial composition and decreased bacterial diversity of the fecal microbiota in AD patients compared with healthy elderly subjects. The structural dysbiosis of the fecal microbiota of the AD patients was characterized by reductions in butyrate-producing bacteria, such as *Faecalibacterium*, and increases in lactate-producing ones, such as *Bifidobacterium*, which were both significantly correlated with host pro- and anti-inflammatory cytokines as well as clinical indicators of AD in the host. These changes in key functional bacteria, such as the *Faecalibacterium* and *Bifidobacterium*, can be used as non-invasive biomarkers to discriminate between healthy elderly subjects and AD patients. Transformations of the gut microbiota from lactate producers into butyrate producers through personalized diet or intervention from beneficial microbiota may be useful for patient-tailored early intervention in cases of AD. In addition, the functional dysbiosis in AD-associated fecal microbiota also suggests that the changed fecal microbiota is associated with changed functions and metabolic activities of the patients, which might play vital roles in the pathogenesis and development of AD. Therefore, our investigation of fecal microbiota using a large and confirmed AD cohort provides novel insights into disease pathogenesis, which can provide new avenues for the scientific trajectory of managing neurodevelopmental disorders by modulating the gut microbiome.

## Data Availability Statement

The datasets presented in this study can be found in online repositories. The names of the repository/repositories and accession number(s) can be found in the article/Supplementary Material.

## Ethics Statement

The studies involving human participants were reviewed and approved by the Ethics Committee of Lishui Second People's Hospital (Zhejiang, China). The patients/participants provided their written informed consent to participate in this study.

## Author Contributions

ZL, MZ, XY, and SW conceived and designed the experiments. ZL, MZ, XY, YC, LS, XL, and RJ performed the experiments. ZL, MZ, XY, and LS analyzed the data. ZL, MZ, LS, and SW wrote the paper and edited the manuscript. The final manuscript was read and approved by all authors.

## Conflict of Interest

The authors declare that the research was conducted in the absence of any commercial or financial relationships that could be construed as a potential conflict of interest.

## Supplementary Material

Supplementary Figure 1 Variations in the compositions of fecal microbiota in the Chinese AD patients and the healthy controls. Relative proportions of bacterial phyla in AD patients (*n* = 100) and healthy controls (*n* = 71).

Supplementary Figure 2 Variations in the composition of fecal microbiota in the Chinese AD patients and the healthy controls. Relative proportions of bacterial genera in AD patients (*n* = 100) and healthy controls (*n* = 71).

Supplementary Figure 3 Heatmap of the genus-level taxa in the fecal microbiota of the Chinese AD patients and the healthy controls. The color of the spots in the panel represents the relative abundance (normalized and log_10_ transformed) of the genus in each sample. The relative abundance of the bacteria in each genus is indicated by a gradient of colors from blue (low abundance) to red (high abundance). The taxonomic classifications of the family are shown on the right. The corresponding Shannon's index in each sample is shown under the heatmap.

Supplementary Figure 4 Mean concentrations (pg/ml) of pro- and anti-inflammatory cytokines and chemokines in patients with AD and in healthy controls determined using Bio-Plex immunoassays. The concentrations of TNF-α **(A)** and IP-10 **(D)** increased significantly in patients with AD, while those of IL-8 **(B)**, MCP-1 **(E)**, MIP-1a **(F)**, and IFN-γ **(C)** decreased significantly. ^*^*p* < 0.05.

## Abbreviations

ACE: abundance-based coverage estimator
AD: Alzheimer's disease
AUC: an area under the curve
Aβ: amyloid-β
BMI: body mass index
CNS: central nervous system
FDR: false discovery rate
F/B: *Faecalibacterium*/*Bifidobacterium*
IFN-γ: interferon gamma
IL: interleukin
IP-10: interferon gamma-inducible protein 10
KEGG: Kyoto Encyclopedia of Genes and Genome
LDA: linear discriminant analysis
LEfSe: linear discriminant analysis effect size
MCP-1: monocyte chemotactic protein-1
MIP: macrophages inflammatory protein
MMSE: Mini-Mental State Examination
MRI: magnetic resonance imaging
no: numbers
OTU: operational taxonomic unit
PCoA: principal coordinate analysis
PiCRUSt: phylogenetic investigation of bacterial communities by reconstruction of unobserved states
QIIME: Quantitative Insights Into Microbial Ecology
RDP: Ribosomal Database Project
SCFAs: short chain fatty acids
SD: standard deviation
SparCC: sparse compositional correlation
STAMP: Statistical Analysis of Metagenomic Profiles software package
TNF-α: tumor necrosis factor-alpha
WAIS: Wechsler Adult Intelligence Scale.
