# Supplementary figures and images for: Structural and Functional Dysbiosis of Fecal Microbiota in Chinese Patients With Alzheimer's Disease
Source: Front Cell Dev Biol. 2021 Feb 4;8:634069. doi: 10.3389/fcell.2020.634069 (PMC7889981; doi:10.3389/fcell.2020.634069)

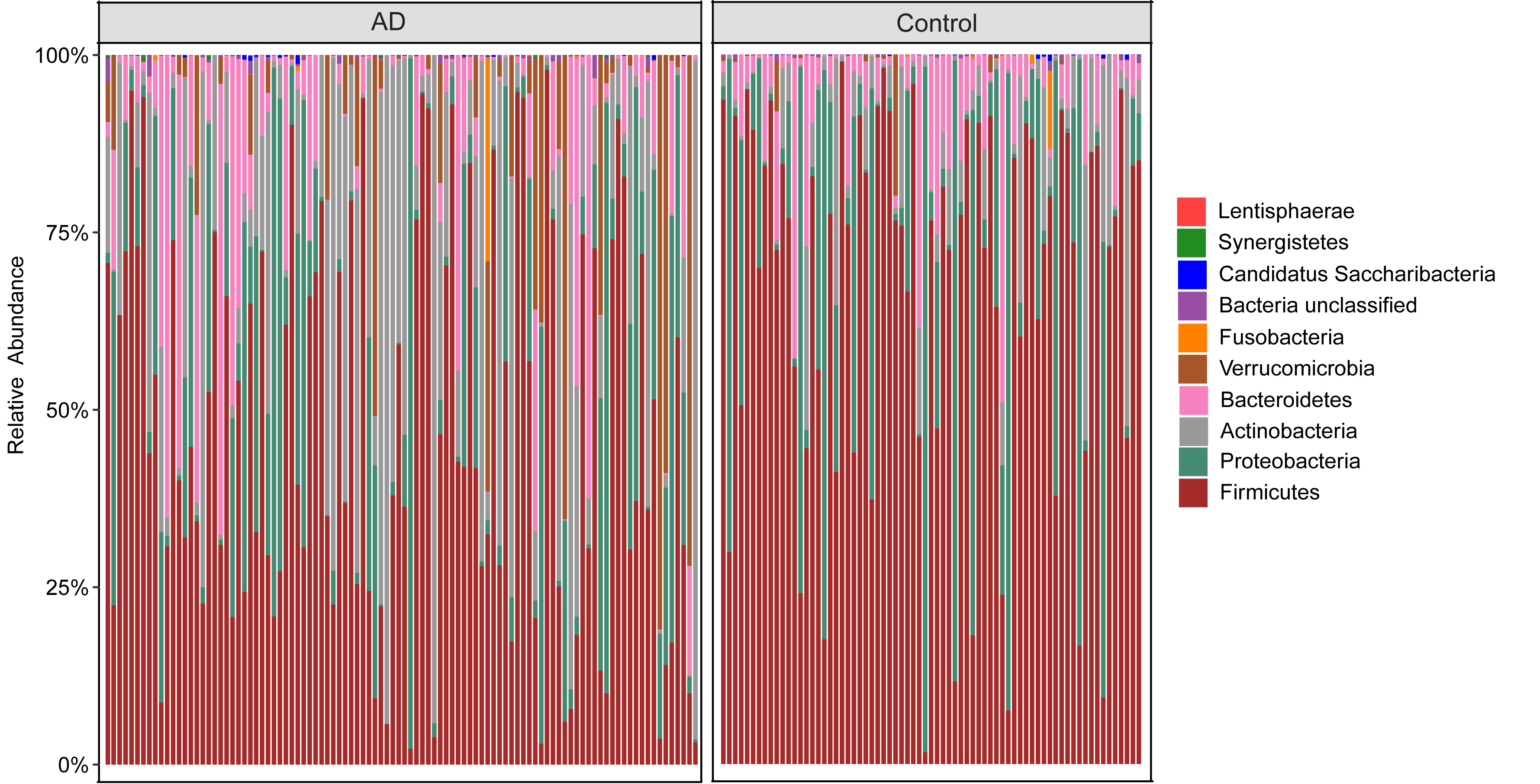

Supplement: Supplementary Figure 1 — Variations in the compositions of fecal microbiota in the Chinese AD patients and the healthy controls. Relative proportions of bacterial phyla in AD patients (n = 100) and healthy controls (n = 71). [file Image_1.TIF]

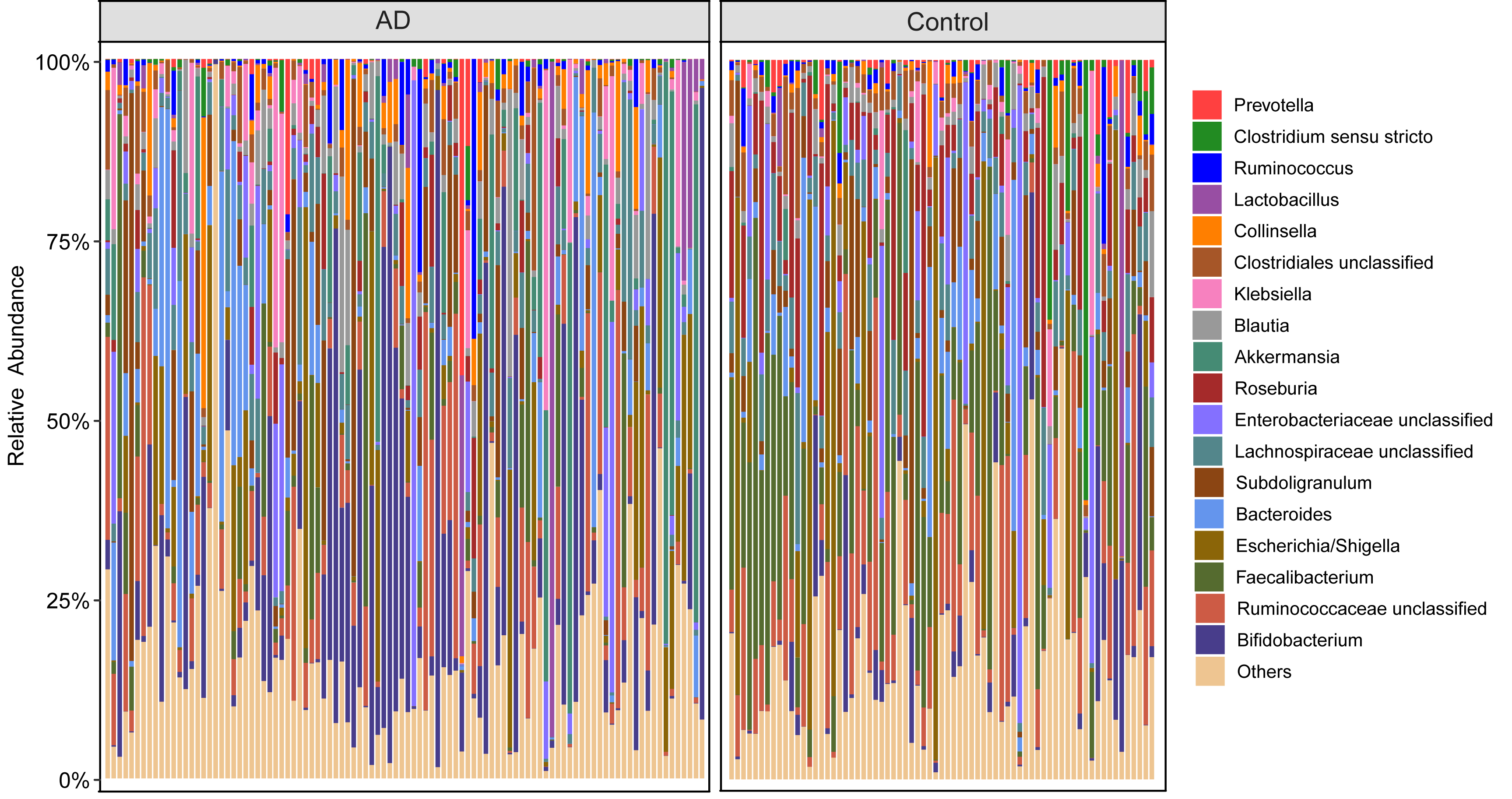

Supplement: Supplementary Figure 2 — Variations in the composition of fecal microbiota in the Chinese AD patients and the healthy controls. Relative proportions of bacterial genera in AD patients (n = 100) and healthy controls (n = 71). [file Image_2.TIF]

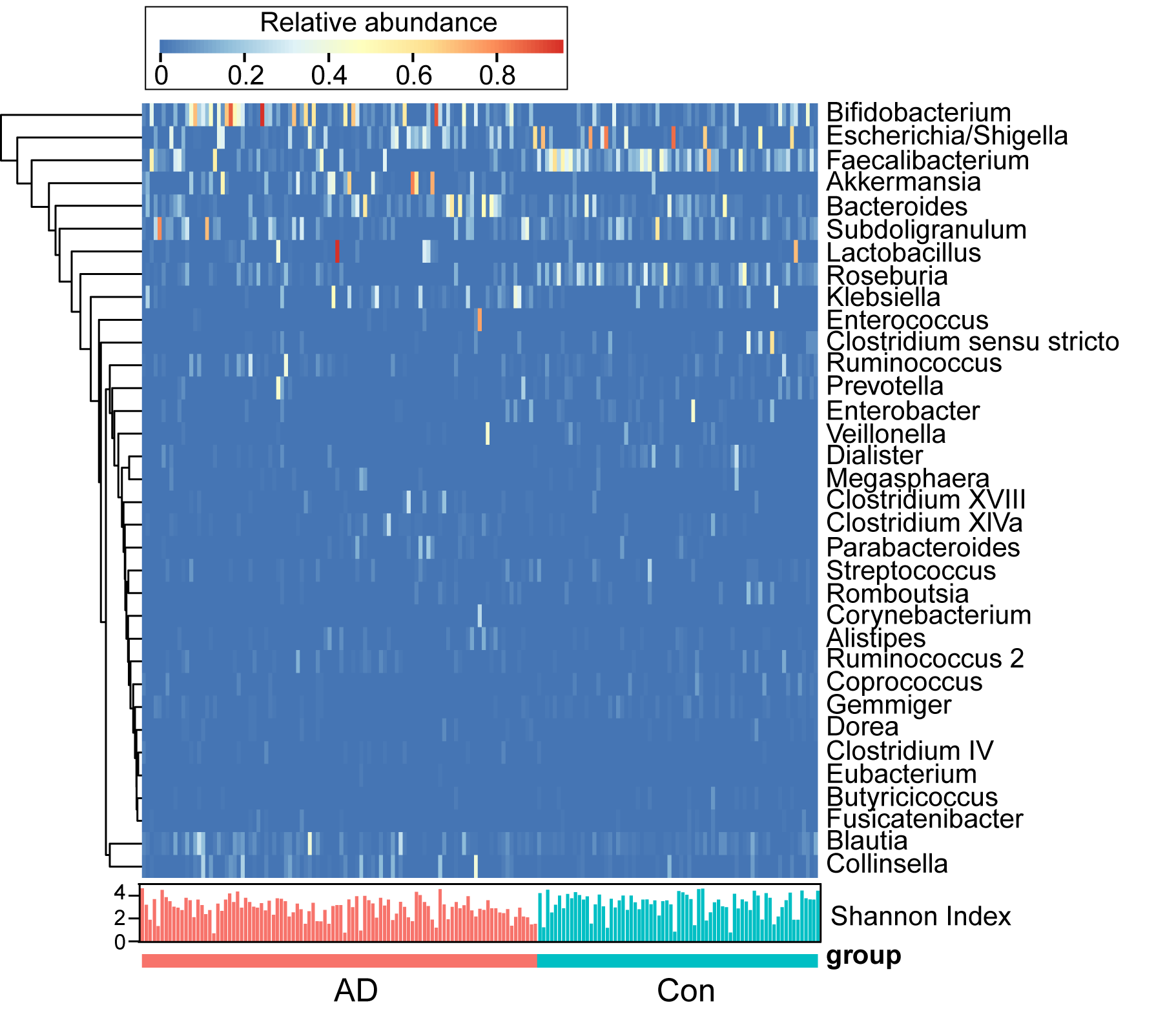

Supplement: Supplementary Figure 3 — Heatmap of the genus-level taxa in the fecal microbiota of the Chinese AD patients and the healthy controls. The color of the spots in the panel represents the relative abundance (normalized and log10 transformed) of the genus in each sample. The relative abundance of the bacteria in each genus is indicated by a gradient of colors from blue (low abundance) to red (high abundance). The taxonomic classifications of the family are shown on the right. The corresponding Shannon's index in each sample is shown under the heatmap. [file Image_3.TIF]

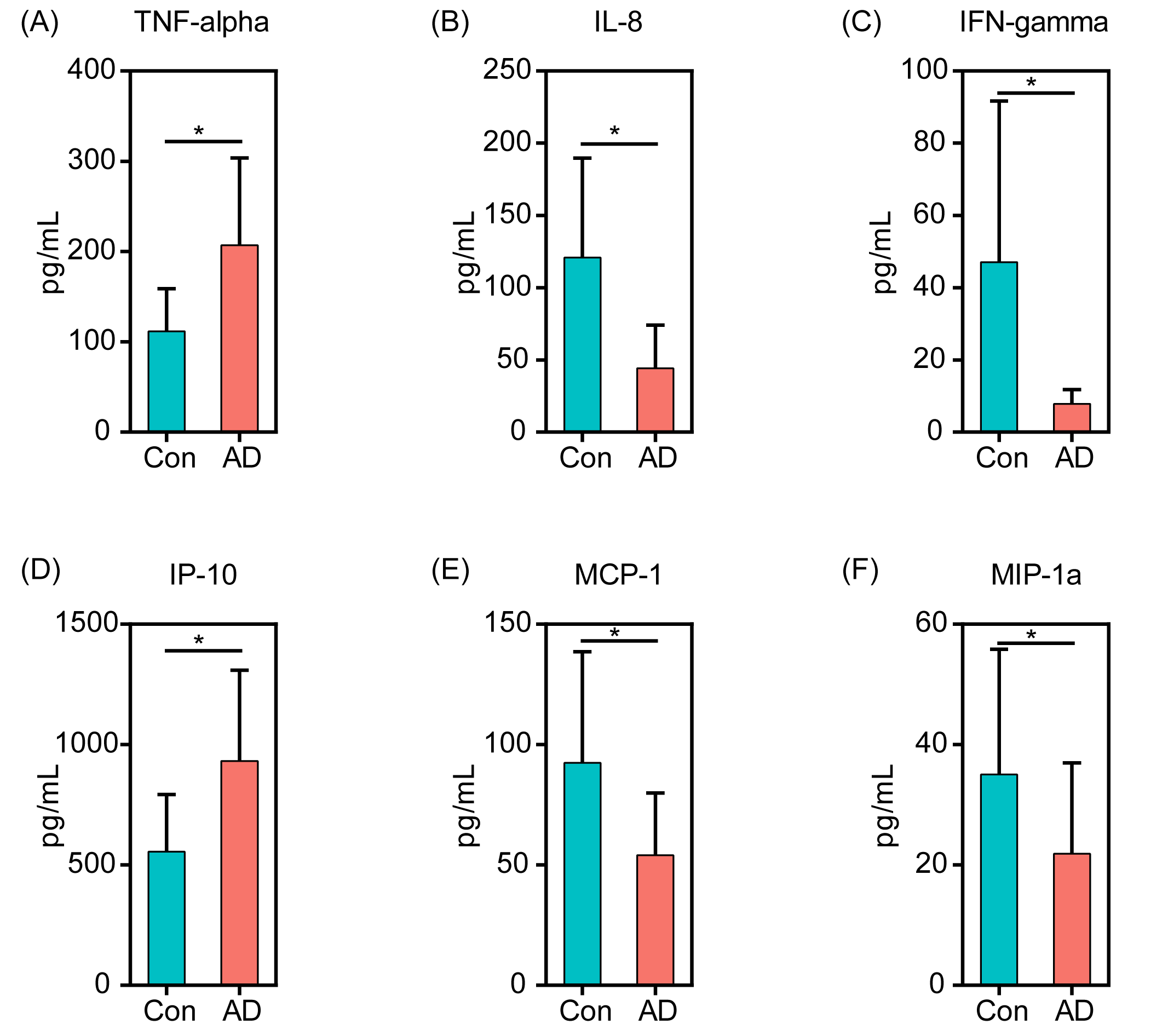

Supplement: Supplementary Figure 4 — Mean concentrations (pg/ml) of pro- and anti-inflammatory cytokines and chemokines in patients with AD and in healthy controls determined using Bio-Plex immunoassays. The concentrations of TNF-α (A) and IP-10 (D) increased significantly in patients with AD, while those of IL-8 (B), MCP-1 (E), MIP-1a (F), and IFN-γ (C) decreased significantly. *p < 0.05. [file Image_4.TIF]
